# Eviction, intimate partner violence and HIV: Expanding concepts and assessing the pathways through which sexual partnership dynamics impact health

**DOI:** 10.1016/j.socscimed.2022.115030

**Published:** 2022-05-11

**Authors:** Allison K. Groves, Patrick D. Smith, Luwam T. Gebrekristos, Danya E. Keene, Alana Rosenberg, Kim M. Blankenship

**Affiliations:** a Dornsife School of Public Health, Drexel University, 3215 Market Street, Philadelphia, 19104, 267 359 6274, USA; b Yale University School of Public Health, Department of Social and Behavioral Sciences, 60 College Street, New Haven, CT, 06510, USA; c American University, Department of Sociology, 4400 Massachusetts Avenue, Washington DC, 20016, USA

**Keywords:** Eviction, Housing instability, Intimate partner violence, HIV prevention, Sexual risk, Mediation, Pathways, Longitudinal

## Abstract

Over 2 million renters in the United States are legally evicted annually, and even more renters experience other landlord-related forced moves each year. While past research has documented an association between legal eviction and HIV risk, no studies have examined the relationship between forced moves and sexual partnership dynamics longitudinally, or the pathways through which forced moves impact such risk. Addressing this gap is imperative, particularly given inequities that place Black renters and women at disproportionate risk of eviction.

This study leverages data from a longitudinal cohort study of 282 adults in New Haven to examine whether landlord-related forced moves reported at baseline (including, but not limited to, legal eviction) is associated with HIV sexual risk reported six months later. We use bootstrapped path analyses to examine intimate partner violence (IPV) victimization and perpetration as potential mediators.

One-fifth of participants (21.2%) had experienced a landlord-related forced move at baseline. At follow up, nearly two-thirds (63.8%) reported at least one HIV sexual risk factor, one in seven (14.2%) reported IPV victimization, and one in ten (10.3%) reported IPV perpetration. Individuals who reported landlord-related forced moves were more likely to report IPV victimization (standardized β = 0.19, SE = 0.08, p = .02) and IPV perpetration (β = 0.25, SE = 0.09, p = .003). Both IPV victimization and perpetration mediated the association between landlord-related forced moves and HIV sexual risk (indirect victimization effect, β = 0.09, SE = 0.05, p = .06; indirect perpetration effect, β = 0.16, SE = 0.07, p = .02), though IPV victimization was only marginally significant.

In conclusion, IPV is itself a negative consequence of forced moves that also contributes to other negative health effects, like HIV risk. Therefore, providers should offer violence screening and referral for clients who have recently faced a forced move. Simultaneously, policy-level solutions to prevent eviction and increase housing affordability are urgently needed to address the rising burden – and inequitable distribution – of evictions among low-income renters.

## 1. Introduction

Housing instability is a known social determinant of poor health in general (Swope and Hernández, 2019; World Health Organization, 2018), and HIV risk, specifically (Corneil et al., 2006; Marshall et al., 2009; Shubert and Bernstine, 2007). Eviction is an acute form of housing instability, and an event that is increasingly common among renters in the United States. More than 2 million individuals in the United States (US) are legally evicted from their housing every year (Eviction Lab, 2022). This eviction crisis, which disproportionately impacts Black females and other non-white renters (Hepburn et al., 2020), has been exacerbated by the COVID-19 pandemic (Eviction Lab, 2022). While a growing literature documents the negative health impacts of legal eviction, fewer studies have examined its link with HIV risk, or assessed whether the association persists when including individuals who are forced to move by their landlord through other means. There is also limited knowledge of the pathways through which eviction and similar types of forced moves may impact HIV risk. Given the very high rates of eviction in the United States, along with gender and race disparities in who is evicted, understanding the impacts and pathways through which landlord-related forced moves operate is key both to developing clinic-based and policy approaches to prevent such moves and to reducing disparities in HIV.

A majority of individuals who are legally evicted are evicted because of non-payment of rent, which itself is tied to a growing unaffordable housing crisis in the US (Aurand et al., 2021; Desmond, 2020). That is, there is currently no state in the US in which a full-time minimum wage job is sufficient to afford a one-bedroom apartment (National Low Income Housing Coalition, 2021). As with inequities in eviction, racial inequities in housing affordability are striking: 54% of Black renters experience at least moderate cost burden, compared to 42% of non-Latinx white renters (Joint Center for Housing Studies of Harvard University, 2021). At the same time, the demand for rental subsidies (i.e., vouchers and/or public housing) exceeds supply: only 1 in 5 eligible households receive such a subsidy, and the waitlists typically average 2 years or more (Center on Budget and Policy Priorities, 2021a).

Landlords may evict tenants for reasons other than non-payment of rent. For instance, they may evict tenants that they suspect of illegal activity, or those who are labeled as a “nuisance” due to frequent 911 calls (Demsas, 2021; Gavin, 2014). Landlords also may use the threat of eviction to limit tenants’ ability to assert rights surrounding housing quality and safety (Garboden and Rosen, 2019). They may use serial eviction filings as a means of collecting late fees associated with non-payment of rent (Leung et al., 2021). And, landlords may file for evictions without cause as a means of forcing out existing renters and attracting a clientele that they perceive as more desirable, particularly in gentrifying neighborhoods (Newman and Wyly, 2006). The intersection of such interests may explain why many legal evictions for non-payment of rent occur in cases where tenants owe an amount less than one month’s rent (Louis et al., 2020).

An insufficiency of affordable housing units relative to demand, along with an array of legislative factors that prioritize the needs of property owners over tenants, contribute to a striking landlord-tenant power differential (Garboden and Rosen, 2019; Gold, 2016). This power differential means that landlords have the ability to force renters out not only through legal eviction, but also through other means. For example, they might threaten to evict tenants, tell tenants they have to leave, or raise the rent instead of pursuing formal eviction. Indeed, in the few studies which have examined eviction more broadly, it appears that legal eviction is just the tip of the iceberg. For example, in one study in Milwaukee, one in eight renters experienced a landlord-related forced move (LRFM) in the past two years, and only a quarter of these renters were legally evicted. In a review of 2017 American Housing Survey data, researchers found that rates of informal evictions exceeded legal evictions by a factor of 5.5 (Gromis and Desmond, 2021). Moreover, in our own research in, New Haven, one in five renters experienced a LRFM in the past two years, yet only 20% of these were the result of legal eviction (Groves et al., 2021).

The physical displacement, material hardship, and stress resulting from eviction has significant negative effects across the life course (Vásquez-Vera et al., 2017). Eviction is associated with adverse birth outcomes (Himmelstein and Desmond, 2021). Children whose families/households experience an eviction are at increased risk of adverse social outcomes, including greater food insecurity (Leifheit et al., 2020) and higher rates of school exit (Haley, 2020). For adolescents and young adults, eviction is associated with worse self-rated health, general health and mental health (Hatch and Yun, 2020; Hoke and Boen, 2021). For low-income mothers, eviction is associated with material hardship, worse self-rated health, depression, and parenting stress (Desmond and Kimbro, 2015).

Emerging evidence also suggests that the physical displacement, material hardship, and stress from an eviction may affect sexual partnership dynamics commonly associated with HIV acquisition, including intimate partner violence (IPV). That is, in accordance with theories of stress (Goodman et al., 2009; Lucero et al., 2016), housing instability caused by a landlord-related forced move may lead to an increase in conflict, and thus, IPV, within a partnership. Specifically, the situational impact of being forced to leave one’s home may override an individual’s coping capacity, or ability to manage their reaction to the stress associated with this situational event, particularly in the absence of sufficient resources. The subsequent powerlessness (both real and perceived) in turn, may increase the likelihood that an individual will act aggressively – and perpetrate violence – against their partner. Likewise, one’s restricted capacity to navigate, respond to, or cope with the stress associated with the loss of one’s home and the subsequent powerlessness resulting from the loss may also increase their susceptibility to violence. Indeed, prior research with a nationally representative sample found recent housing insecurity to be associated with multiple types of IPV among both men and women (Breiding et al., 2017). In research specifically examining the link between eviction and IPV, perpetration of severe physical IPV was significantly higher among those who were evicted than those who were not, and there was no difference in the association by gender (Schwab-Reese et al., 2016). In research conducted among female IPV survivors, receipt of stable housing decreased female IPV survivors’ risk of subsequent violence (Rollins and Billhardt, 2012).

In addition, housing instability (including but not limited to eviction), has been linked to HIV acquisition risk. In a research study with women with a history of criminal legal involvement, those women who reported high housing volatility (i.e., lived in three or more residences in the past six months) were significantly more likely to report unprotected sex and sex exchange than women with low housing volatility (Weir et al., 2007). In our own cross sectional research study with low income men and women, eviction and other landlord-related forced moves were associated with higher odds of unprotected sex, concurrent sex, and transactional sex (Groves et al., 2021). At an ecological level, county-level eviction rates is associated with rates of gonorrhea and chlamydia (Niccolai et al., 2019).

And finally, IPV has significant impacts on sexual health, including HIV. Specifically, a recent meta-analysis which examined links between IPV and HIV found strong evidence that both physical IPV and any type of IPV were associated with increased HIV infection among women (Li et al., 2014). IPV is a common occurrence among individuals in the U.S, such that 1 in 10 men (10.1%) and 1 in 4 women (25.1%) report experiencing at least 1 type of IPV in their lifetime (Smith et al., 2015), and approximately 1 in 20 men (5.2%) and 1 in 18 women (5.5%) report experiencing at least 1 type of IPV in the past year. Per theoretical and empirical research, violence within a relationship may impact HIV risk directly (e.g., forced sex with a partner living with HIV may increase transmission risk) and indirectly (e.g., violence may decrease one’s ability to negotiate the circumstances surrounding sex, including condom use (Dunkle and Decker, 2013; Maxwell et al., 2015), which in turn, may increase transmission risk).

The growing literature on eviction and sexual partnership dynamics, while important, suffers from three limitations. First, to our knowledge, there are no longitudinal studies which assess the association between landlord-related forced moves and sexual risk. Establishing temporality is of value given that HIV risks like IPV and/or transactional sex may also increase an individual’s risk of eviction. Indeed, nearly all existing research that has documented the link between eviction and IPV or between eviction and HIV sexual risk is descriptive in nature. Second, while housing instability has been linked to IPV, and IPV is a known risk factor for HIV acquisition (Li et al., 2014), no studies have examined whether IPV is a mechanism through which landlord-related forced moves impact HIV risk. And finally, past research on the link between eviction and health has consistently relied on more narrowly defined measures of eviction (Hazekamp et al., 2020; Himmelstein and Desmond, 2021; Niccolai et al., 2019), which frequently exclude those landlord-related forced moves that occur outside the legal system. Like legal evictions, landlord-related forced moves constitute significant disruptions in housing and are thus likely to affect HIV risk in similar ways.

The current study attempts to fill these gaps in the literature. In particular, we expand understanding of the impact of landlord-related forced moves on HIV risk by examining the association prospectively, by using a more comprehensive measurement of eviction than past studies, and by investigating whether IPV is one pathway through which such involuntary displacement may affect dynamics within sexual partnerships.

## 2. Methods

### 2.1. Study design and population

The analytic sample for the current analysis is drawn from participants in the Justice, Housing and Health Study (JustHouHS), which is a longitudinal study conducted in New Haven, Connecticut and designed to examine how mass incarceration and housing instability intersect to impact sexual practices. New Haven’s legal eviction rate was over 4% in 2016, which is the 69th highest eviction rate of large cities in the nation, and considerably higher than the national eviction rate (2.3%) (Eviction Lab, 2022; Hepburn et al., 2020). Like other cities throughout the U.S, New Haven faces a growing housing affordability crisis; fair-market rent for a one-bedroom apartment is $1,181, nearly twice as high as the rent ($676) considered affordable at minimum wage of $13/hour.

Participants were recruited for the JustHouHS cohort using a combination of flyers, outreach to local service providers, community meetings, and snowball sampling. Interested participants (n = 616) were screened by research staff. Of those eligible (n = 471), 71 individuals did not enroll into the study. Participants were eligible for JustHouHS if (1) they were 18 years or older and a resident of New Haven, (2) no household members were already in the study, and (3) they met one of the following criteria: (a) received housing or food assistance in the past year, (b) were Medicaid recipients, (c) were homeless, or (d) resided in low-income census tracts (i.e., more than 20% of residents lived below the federal poverty level). The total sample was also stratified to include 200 individuals released from prison or jail in the past year and 200 who were not recently released (though may have had a history of incarceration). All participants provided consent to participate. JustHouHS participants completed a self-administered computer-assisted survey at each study visit and were compensated $50 for their time at each visit. All study activities were approved by the Yale University IRB board, which served as the IRB of record.

A total of 400 participants enrolled in JustHouHS. For the current analyses, we used participants’ baseline survey data (collected between September 2017–March 2018) and participants’ follow-up 1 survey data (collected between April–August 2018). Each participant completed the follow-up survey 6 months after the baseline survey (mean 6.5 months (sd:0.6)). We restricted the analysis to these two data points because we were specifically interested in examining the short-term consequences of a landlord-related forced move on sexual partnership dynamics. We excluded participants who: (a) did not complete follow-up 1 (n = 82); (b) were HIV-positive at baseline (n = 31); or (c) were missing data on key variables (n = 5), yielding an analytic sample of 282 participants. All participants who self-identified as living with HIV at baseline reported they had been diagnosed more than two years prior to the baseline survey.

### 2.2. Measures

#### 2.2.1. Exposure

Consistent with our prior conceptualization (Groves et al., 2021), participants were coded as having a landlord-related forced move at baseline if: (1) they reported that they had been legally evicted in the past two years; and/or (2) they reported that, in the past 2 years, their last move had occurred for any of the following reasons: I was evicted, the landlord raised the rent, I was forced to move because of non-payment of rent, I was forced to move because of damage to rental unit, I was forced to move because I was accused of illegal drug activities (including sale and/or use), I was forced to move because I was accused of illegal activity (not drug related), I was forced to move because landlord said there were too many people living there, or I was forced to move because the landlord went into foreclosure.

#### 2.2.2. Outcome

The HIV sexual risk outcome, assessed at follow up 1, is based on measures commonly used to examine HIV risk in other studies (Doherty et al., 2000; Drumright et al., 2004; Eshleman et al., 2011; Jenness et al., 2011; Patel et al., 2014; Rucinski et al., 2020; Warner et al., 2006; Zhan et al., 2011). We created a binary measure for HIV sexual risk based on participants’ responses to questions on unprotected sex, concurrency, perceived partner concurrency, and transactional sex. All participants were asked if they had had any unpaid sexual partners in the past six months. If they responded “yes”, they then answered a series of questions about their sexual behavior with each of these partners for up to four sexual partners. (1) *Unprotected sex* Individuals who reported “always using condoms” or who reported “no sexual partners” in the past six months received a ‘0’ for unprotected sex; all others received a ‘1’. (2) *Concurrency* Individuals who responded “yes” to “during the same time period that you were having sex with [partner X], in the last 6 months, were you also having sex with anyone else?” for any sexual partner received a ‘1’ for concurrency; individuals who responded “no” for each sexual partner or who reported no sexual partners received a ‘0’. (3) *Perceived partner concurrency* Individuals who suspected that any partner had another sexual partner received a ‘1’ for perceived partner concurrency. Individuals who did not suspect that their partner had other sexual partners or reported no sexual partners in the last 6 months received a ‘0’. (4) *Sold sex in exchange for money or drugs* Individuals who responded “no” to sold sex in exchange for money or drugs in the last 6 months received a ‘0’; all others received a ‘1’. (5) *Provided sex in exchange for a place to live in the last six months* Finally, in a series of questions about their housing arrangements, participants were asked if, in the last 6 months, they had provided sex in exchange for a place to stay. Individuals who reported “no” received a ‘0’; all others received a ‘1.’ Participants who received a ‘1’ for any of the aforementioned variables were coded as ‘1’ for HIV sexual risk; all others were coded as ‘0.’

#### 2.2.3. Mediators

IPV victimization and IPV perpetration were measured at follow up 1 using 10 items on physical and sexual violence from the WHO modified conflict tactics scale (World Health Organization, 2005). (1) *IPV victimization* Participants who reported having unpaid partners in the past six months were asked about the frequency at which they experienced the 10 acts of violence with each sexual partner for up to four sexual partners. Participants who reported at least 1 act of violence across all partnerships were coded ‘1’; all others received ‘0.’ (2) *IPV perpetration* Participants who reported having unpaid partners in the past six months were asked about the frequency at which they engaged in the 10 acts of violence with each sexual partner for up to four sexual partners. Participants who reported at least 1 act of violence across all partnerships received a ‘1’; all others received a ‘0.’

#### 2.2.4. Covariates

We included the following sociodemographic and contextual variables as potential confounders of the association: age, gender (male, female), race/ethnicity (Black, white, other), education (less than high school, high school, more than high school), individual income in the last month (reported in dollars), ever had a mental health diagnosis (yes/no), drug use in the past 30 days (yes/no), injection drug use in the past 30 days (yes/no), heavy alcohol use (drank more than 15 days) in the past 30 days (yes/no), and incarceration in the past two years (yes/no). Further, we controlled for the number of weeks between baseline and follow-up (in weeks).

### 2.3. Analyses

In our analyses, we first examined sociodemographic and contextual characteristics of our sample and used Chi-square and *t*-tests to determine whether such characteristics were significantly associated with landlord-related forced moves and IPV. Next, we assessed in separate models whether IPV victimization or IPV perpetration mediated the pathway between landlord-related forced moves and HIV sexual risk using path analysis. Sociodemographic and contextual variables that were significantly associated (p < .05) with IPV and HIV sexual risk were included in the path analyses as covariates. We assessed goodness of fit for mediation models using the following indices: CFI and TLI >0.95 and RMSEA <0.05 (Hu and Bentler, 1999). To assess the statistical significance of the indirect effects, we used bootstrapping of 5000 samples (Preacher and Hayes, 2004). All analyses were conducted using R Studio (Rosseel, 2012; RStudio Team, 2021).

We conducted sensitivity analyses to investigate whether the findings changed if we varied the construction of the HIV sexual risk outcome. Specifically, we examined mediation models where HIV sexual risk was constructed as a latent variable (rather than a binary variable). In these models, the pattern of findings was similar. We did not examine mediation models for each separate HIV sexual risk outcome because we did not have enough power to do so for all of the outcomes of interest.

## 3. Results

### 3.1. Descriptive statistics

Table 1 illustrates the study participants’ sociodemographic and contextual characteristics at baseline. Participants’ mean age was 44.9 (sd:11.5). Nearly two-thirds of participants identified as Black (64.9%), and more than one-third of participants were female (35.5%). One in five participants had less than a high school education (20.9%) and the mean monthly income reported was $1070 (sd:$5970). At baseline, approximately one in ten participants (11.0%) reported recent heavy alcohol use, and just over one quarter (26.2%) reported recent drug use.

At baseline, more than one-fifth of participants (n = 60/282) reported a landlord-related forced move in the past two years (Table 1: 21.1%), of which only 21% were the result of a legal eviction (n = 13/60). Participants who reported landlord-related forced moves were like those participants who did not across most sociodemographic and contextual characteristics, apart from age and mental health diagnosis. Participants who reported landlord-related forced moves were significantly younger than those who did not (42.2 vs 45.7, p = .04). Further, participants who reported landlord-related forced moves were more likely to ever have had a mental health diagnosis (68.3% vs 51.4%, p = .02).

At follow up, nearly two-thirds of participants reported HIV sexual risk within the last six months (Table 1: 63.8%). Over half reported unprotected sex (59.2%), and approximately one in five reported concurrency within their sexual partnership (either that they were concurrent (19.9%), or that they suspected their partner was concurrent (22.0%); data not shown). Fewer participants reported exchanging sex for money or drugs (7.1%) or for a place to live (2.1%; data not shown). While HIV risk was higher among individuals who reported a landlord-related forced move as compared to those who did not (71.7% vs 61.7%, respectively), the difference was not statistically significant in bivariate analysis (p = .15).

Also at follow up, nearly one in seven participants (Table 1: 14.2%) reported IPV victimization and one in ten (Table 1: 10.3%) reported IPV perpetration in the last six months. IPV victimization and perpetration were highly correlated (r = 0.91). Participants who had experienced a landlord-related forced move were more likely to report IPV victimization compared to those who did not (25.0% vs 11.3%, p = .007). Similarly, participants who had experienced a landlord-related forced move were more likely to report IPV perpetration compared to those who did not (21.7% vs 7.2%, p = .0001).

Moreover, as seen in Table 2, participants who reported IPV at follow up were like those who did not report IPV across all sociodemographic and contextual characteristics at baseline, aside from recent heavy alcohol use and recent drug use. Participants who reported recent heavy alcohol use at baseline were more likely to report IPV perpetration (25.8% vs 8.3%, p = .007). Participants who reported recent drug use at baseline were more likely to report IPV victimization (21.6% vs 11.5%, p = .03, respectively). Finally, as also seen in Table 2, HIV risk was higher among individuals who had experienced IPV victimization as compared to those who did not (90.0% vs. 59.5%, p = .0001), and among those who had reported IPV perpetration as compared to those who did not (96.6% vs. 60.1%, p=<.0001). Only recent drug use was associated with HIV risk and therefore was included in the models as a covariate.

### 3.2. Path analyses

**Fig. 1.**
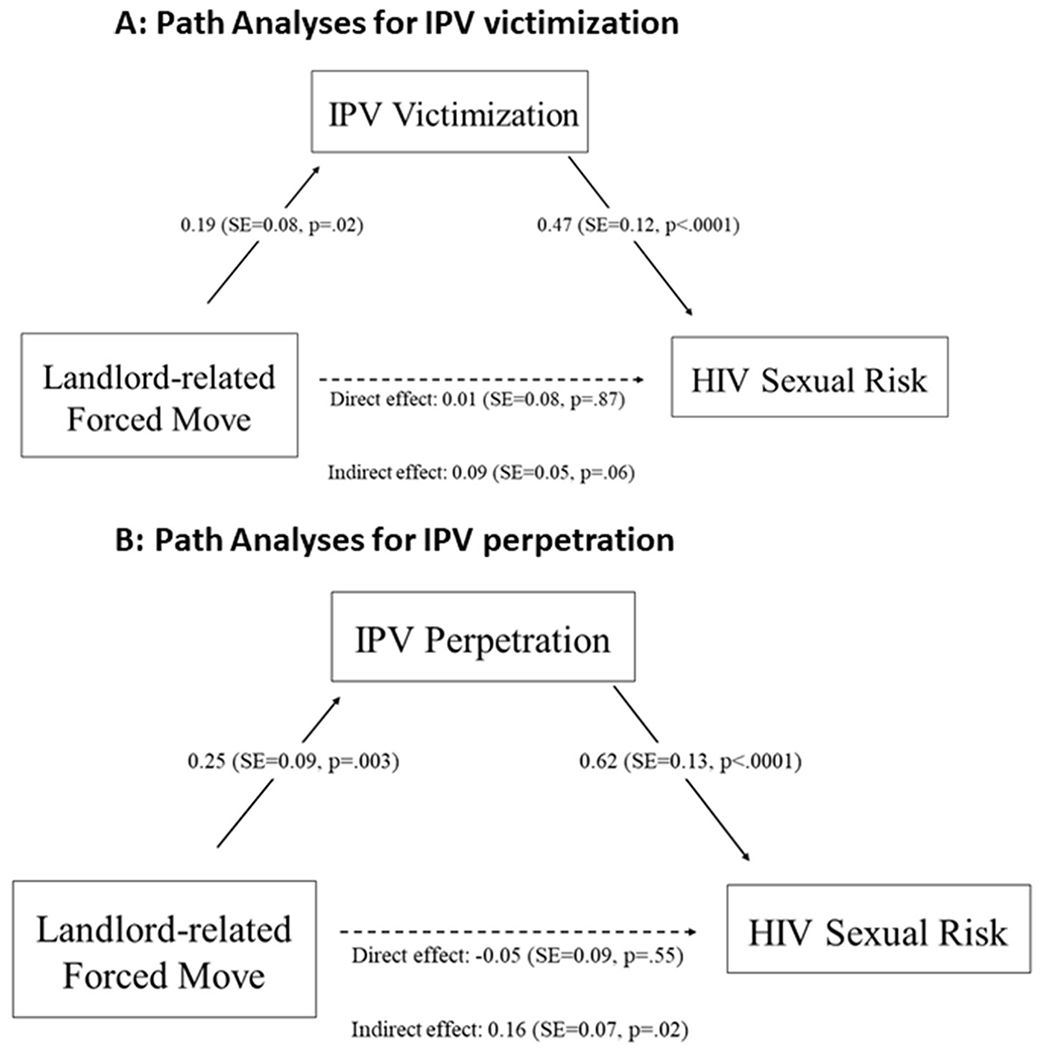
A) Path Analyses for IPV victimization, B) A) Path Analyses for IPV perpetration.

Fig. 1A shows the results from the path analyses examining IPV victimization as the mediator between landlord-related forced moves and HIV risk. The model showed a good fit (CFI = 1.00, TLI = 1.00, RMSEA = 0.000). Reporting a landlord-related forced move was positively associated with IPV victimization (standardized β = 0.19, SE = 0.08, p = .02), meaning that individuals who reported a landlord-related forced move were more likely to report IPV victimization. Further, IPV victimization was directly and positively associated with HIV sexual risk (standardized β = 0.47, SE = 0.12, p < .0001). Finally, landlord-related forced moves marginally influenced HIV sexual risk through IPV victimization (standardized β = 0.09, SE = 0.05, p = .06) (Fig. 1A).

Fig. 1B shows the results from the path analyses examining IPV perpetration as the mediator between landlord-related forced moves and HIV risk. The model also showed a good fit (CFI = 1.00, TLI = 1.00, RMSEA = 0.000). Reporting a landlord-related forced move was positively associated with IPV perpetration (standardized β = 0.25, SE = 0.09, p = .003). IPV perpetration was directly and positively associated with HIV sexual risk (standardized β = 0.62, SE = 0.13, p < .0001). Finally, landlord-related forced moves indirectly influenced HIV sexual risk through IPV perpetration (standardized β = 0.16, SE = 0.07, p = .02) (Fig. 1B).

## 4. Discussion

The impact of eviction and other landlord-related forced moves on HIV risk has been underexplored in existing research. The objective of the current study was to examine whether landlord-related forced moves wwere associated with HIV sexual risk, and to assess whether IPV victimization and IPV perpetration mediated this risk. While we did not find evidence of a direct effect between landlord-related forced moves and HIV risk, we found that individuals who reported a landlord-related forced move subsequently had higher levels of IPV in their sexual partnerships than those who did not. Further, we found that higher levels of IPV were associated with increased HIV sexual risk. These findings—that landlord-related forced moves affected HIV sexual risk via heightened IPV – add to a growing body of evidence that the disruptions caused by landlord-related forced moves negatively impact health.

IPV is a leading cause of housing instability (Chan et al., 2021; Dillon et al., 2016; Montgomery et al., 2018). That is, an individual experiencing IPV may feel that they have no choice but to leave the house for their own safety and subsequently face challenges securing and/or accessing stable housing in the future (Daoud et al., 2016). Indeed, women who experienced psychological or physical IPV in a longitudinal cohort study in the United Kingdom were at increased risk of incident homelessness compared to women who had not (Chan et al., 2021), and there are other cohort studies in the U.S. and Australia which also show higher levels of housing instability among women who have prior experiences of IPV (Dillon et al., 2016; Montgomery et al., 2018). In addition, descriptive research suggests that when an individual who is experiencing IPV makes frequent calls to 911, they risk being labeled as a “common nuisance,” which can result in eviction from the property (Desmond and Valdez, 2013; Gavin, 2014).

And yet, as reflected in our study, IPV may also be a consequence of eviction. To the best of our knowledge, our study is the first longitudinal study to provide evidence that landlord-related forced moves lead to increased IPV within sexual partnerships. These findings are aligned with one other longitudinal study, in which eviction was associated with increased risk of any type of violence, which may have been inclusive of but was not restricted to IPV (Kennedy et al., 2017). Future research might build on these collective findings to identify which protective factors weaken the association between landlord-related forced moves and IPV. For example, it may be that individuals who have stronger support networks (beyond their sexual partner) or greater resources (including access to emergency housing) are buffered from some of the stress which accompanies the unwanted move. On the other hand, future research may also assess whether the negative impacts of landlord-related forced moves on IPV are worse among individuals who have pre-existing vulnerabilities or who experience other co-occurring stressors along with the landlord-related forced move.

Similarly, research is needed to assess whether landlord-related forced moves affect IPV differently for different groups of individuals. We did not have the statistical power to assess whether the relationship varied by gender. Nevertheless, we did not observe an association between gender and IPV victimization, which contradicts research findings on victimization using national datasets, in which women face higher risk of both physical IPV victimization and sexual IPV victimization than men (Smith et al., 2015). It is possible that our findings are limited by the characteristics of our sample population or by the small proportion of women included in the sample. Given the limitations of convenience sampling, as well as the heightened risk that women face for both eviction and IPV (Hepburn et al., 2020; Smith et al., 2015), further research is warranted to determine whether the effects of eviction on IPV victimization differ based on gender.

Understanding how IPV links landlord-related forced moves and HIV sexual risk is particularly important given that we found no direct effect between landlord-related forced moves and HIV risk. The lack of a direct effect in our findings differs from existing literature, in which housing instability generally, and legal eviction specifically, are positively associated with HIV risk (Niccolai et al., 2019; Weir et al., 2007). There are several potential possibilities for conflicting findings. First, it may be that legal eviction impacts HIV risk differently than landlord-related forced moves more broadly. While we did not have the power to examine the impacts of these outcomes separately (e.g., only 13 individuals in our sample reported *only* a legal eviction), future research with larger samples should examine whether there are differential impacts for different forms of landlord-related forced moves. Second, we may not have seen evidence of a direct effect because it is possible that the effect of landlord-related forced moves on HIV risk differs by subpopulation (e.g., the impact of landlord-related forced moves on HIV risk may be different among households with lower pre-existing conflict than households with higher pre-existing conflict). Future research with a representative sample is needed to examine whether the link between landlord-related forced moves and HIV risk is moderated by pre-existing household conflict.

Nonetheless, our findings increase understanding of one mechanism through which landlord-related forced moves impact HIV risk: IPV. Understanding the social mechanisms through which eviction impacts health is critical to effectively responding to and supporting individuals going through such unwanted moves. It is also likely there are other pathways through which landlord-related forced moves may impact HIV acquisition risk; these potential pathways might be explored in future research. For example, landlord-related forced moves may decrease economic stability, which might increase specific sexual behaviors, like transactional sex. In addition, landlord-related forced moves may negatively impact mental health and substance use, which in turn, may compromise an individual’s ability to engage in safe sexual practices. Simultaneously, the displacement caused by a forced move may disrupt one’s access to resources, including medical care and social service providers, which may further exacerbate one’s risk of HIV. And finally, landlord-related forced moves may increase homelessness (Desmond et al., 2015), which in turn may also negatively impact sexual risk. Further research is needed to unpack the complex relationship between the social and economic consequences of landlord related-forced moves and health.

Findings of this study, examined alongside the broader and growing literature on eviction and health, have the potential to inform clinical and policy responses to landlord-related forced moves. Specifically, health and social service providers can work to incorporate standardized screening for housing disruptions (including eviction and other landlord-related forced moves) into routine and episodic care encounters, alongside screeners for other social needs. Such efforts are needed to improve screening within hospitals and outpatient practices, especially in light of a 2018 survey which found that just 24% of hospitals and 16% of outpatient practices routinely screen for patients’ experience with interpersonal violence and their social needs pertaining to food, housing, utilities, and transportation (Fraze et al., 2019). In working to integrate universal screening for social needs, clinicians should be aware of racial and gender disparities in eviction rates. Moreover, they should be cognizant of the heightened risk of IPV that may follow a landlord-related forced move, and of the ways that this IPV risk may impact clients’ sexual risk. For individuals who have experienced – or are at risk for – a landlord-related forced move, providers might follow up to assess relationship conflict and risk of IPV, support safety planning, and connect patients to resources – including social work and/or legal services – that may promote their housing stability or their safety within the context of their sexual relationship. Efforts to improve screening are particularly necessary in the context of the COVID-19 pandemic, during which researchers have observed high rates of material strain and an increase, relative to pre-pandemic levels, in IPV-related calls to police departments (Boserup et al., 2020; Center on Budget and Policy Priorities, 2021b).

In addition, a variety of policy initiatives have the potential to reduce evictions or their long -term consequences, or to address the shortage of affordable housing. While access to legal counsel for tenants has been shown to reduce the rate of evictions in several jurisdictions, estimates suggest that as many as 90% of tenants undergoing legal eviction proceedings lack access to such counsel, placing them at disproportionate risk of an eviction judgment (Boston Bar Association Task Force on the Civil Right to Counsel, 2012; Holl et al., 2016; Sandefur, 2010). In cases where eviction is necessary, implementation of “just cause” policies and post-eviction measures (such as the sealing of eviction records) can lessen discrimination in eviction filings and prevent an accumulation of housing disadvantage for evicted tenants (Benfer et al., 2020). In addition, to ease cost burdens faced by low-income renters and reduce eviction filings, there is a need for further investment in interventions that increase the stock of affordable housing (e.g., through creation of new housing units and renovation of existing uninhabitable units) and make existing units more affordable (e.g., through housing voucher programs, inclusionary zoning policies, and expansion of alternative land ownership models, such as community land trusts). Such interventions are urgently needed, especially considering research which indicates that millions are at risk of eviction and other forced moves following the withdrawal of the federal eviction moratorium. Because Black renters, and especially Black women, face a disproportionately high risk of eviction and other forced moves, policy intervention related to eviction prevention have direct implications for health equity.

This study has several limitations. First, it is based on a convenience sample of low-income individuals, with rates of IPV that are higher than the national average, of whom more than half had a history of criminal legal involvement. Therefore, our findings may not be generalizable. However, while our sample was deliberately not representative with respect to incarceration histories, it is important to note that criminal legal involvement is common in low-income neighborhoods. In fact, 38.5% of the participants who were not recruited based on recent incarceration history reported prior felony convictions. Relatedly, despite existing evidence of inequities in both eviction and HIV by race and gender, we were underpowered to analyze whether the impacts of landlord-related forced moves affected IPV and HIV risk differently by race or gender. At the same time, even if the impacts of landlord-related forced moves are the same across groups, the inequitable distribution of eviction (by race and gender) means that more people of color and women will face associated HIV risk. Therefore, while future research with larger datasets might further understanding of inequities in processes and outcomes by race and gender, our findings still have implications for advancing health equity through policies and programs that seek to reduce eviction. Third, while this study includes a more comprehensive measure of landlord-related forced moves than past studies, we were unable to examine whether the total number of forced moves within the past two years was associated with greater risk of IPV or sexual risk. Fourth, it is possible that previous IPV exposure contributed to landlord-related forced moves at baseline. However, given that IPV at baseline was assessed in the past 6 months and landlord-related forced moves at baseline was assessed for the past 2 years, we are unable to examine the reverse association (e.g., IPV as exposure and landlord-related forced moves as mediator). Relatedly, although utilizing longitudinal data allows us to examine the association between landlord-forced moves and subsequent IPV exposure, we cannot determine causality. Finally, the reference period for whether an individual had experienced a landlord-related forced move at baseline was broad: individuals could have reported such a move at any point in the past 2 years. Given variability across individuals in the time in which they experienced the forced move, it is possible that IPV occurred after the forced move but before the follow-up survey in which we measured IPV. Thus, our inability to infer when in the two-year period an individual experienced a forced move constraints understanding of whether the IPV was an immediate or a longer-term consequence of the forced move. Furthermore, we may have underestimated the strength of the association between landlord-related forced moves and IPV, given the wide period in which forced moves occurred.

## 5. Conclusions

A growing body of literature indicates that eviction - which disproportionately affects women of color – contributes to health inequities. However, the relationship between eviction, IPV, and HIV sexual risk has been surprisingly underexplored in extant literature. Moreover, most existing studies focus on the health impacts of legal eviction even though landlords may force tenants out through other means as well. Our study broadens the conceptualization of landlord-related forced moves and extends understanding of their impacts. Specifically, our findings highlight that legal eviction only represents a portion of those individuals who experience a landlord-related forced move. Our findings also reveal IPV as a negative consequence of such moves, that in turn, contributes to HIV-related sexual risk. To better identify and support individuals at risk of violence and HIV, health and social service providers should incorporate screening for both legal evictions and other forms of landlord-related forced moves into existing workflows, be cognizant of the ways that such forced moves may negatively impact sexual partnerships, and finally, provide referrals and support to individuals who have recently experienced or are at risk of experiencing a forced move. Simultaneously, to address the rising burden of evictions among tenants and to decrease racial and gender inequities in eviction, policymakers should implement and evaluate evidence-based solutions – including right to counsel legislation and housing voucher programs – that can reduce the incidence of evictions and increase the availability of safe, stable, and affordable housing.

## Figures and Tables

**Table 1:** Differences in HIV sexual risk, IPV and baseline characteristics by forced move (N = 282).

	All participants	No Forced Move n = 222 (78.7%)	Forced Move n = 60 (21.3%)	p-value^a^
		N (%)^b^ or Mean (SD)		
*Outcome*				
HIV Sexual Risk				
Yes	180 (63.8%)	137 (61.7%)	43 (71.7%)	0.15
No	102 (36.2%)	85 (38.3%)	17 (28.3%)	
*Mediators*				
IPV Victimization				
Yes	40 (14.2%)	25 (11.3%)	15 (25.0%)	0.007
No	242 (85.8%)	197 (88.7%)	45 (75.0%)	
IPV Perpetration				
Yes	29 (10.3%)	16 (7.2%)	13 (21.7%)	.0001
No	253 (89.7%)	206 (92.8%)	47 (78.3%)	
*Sociodemographic and contextual characteristics*				
Age	44.9 (11.5)	45.7 (11.3)	42.2 (11.9)	0.04
Sex				
Male	182 (64.5%)	147 (66.2%)	35 (58.3%)	0.26
Female	100 (35.5%)	75 (33.8%)	25 (41.7%)	
Race				
Black	183 (64.9%)	144 (64.9%)	39 (65.0%)	0.92
White	72 (25.5%)	56 (25.2%)	16 (26.7%)	
Other	27 (9.6%)	22 (9.9%)	5 (8.3%)	
Income in the last month (in dollars)	1070 (5970)	1110 (6580)	911 (2810)	0.73
Education				
Less than high school	59 (20.9%)	49 (22.1%)	10 (16.7%)	0.58
High school	136 (48.2%)	104 (46.8%)	32 (53.3%)	
More than high school	87 (30.9%)	69 (31.1%)	18 (30.0%)	
Recent heavy alcohol use (30 days)				
Yes	31 (11.0%)	21 (9.5%)	10 (16.7%)	0.11
No	251 (89.0%)	201 (90.5%)	50 (83.3%)	
Recent drug use (30 days)				
Yes	74 (26.2%)	53 (23.9%)	21 (35.0%)	0.08
No	208 (73.8%)	169 (76.1%)	39 (65.0%)	
Recent injection drug use (30 days)				
Yes	6 (2.1%)	3 (1.4%)	3 (5.0%)	0.11
No	276 (97.9%)	219 (98.6%)	57 (95.0%)	
Recent incarceration (past 2 years)				
Yes	141 (50.0%)	109 (49.1%)	32 (53.3%)	0.66
No	141 (50.0%)	113 (50.9%)	28 (46.7%)	
Have a mental health diagnosis				
Yes	155 (55.0%)	114 (51.4%)	41 (68.3%)	0.02
No	127 (45.0%)	108 (48.6%)	19 (31.7%)	
Time lapse between survey (in weeks)	25.8 (2.25)	25.9 (2.25)	25.6 (2.25)	0.32

a For categorical variables, p-values are from χ^2^ tests. For continuous variables, p-values are from t-tests.

b Percentages present are column percentages.

**Table 2:** Differences in HIV sexual risk and baseline characteristics by IPV victimization and perpetration (N = 282).

	All participants	No IPV victimization n = 242 (85.8%)	IPV victimization n = 40 (14.2%)	p-value^a^	No IPV perpetration n = 253 (89.7%)	IPV perpetration n = 29 (10.3%)	p-value^a^
		N (%)^b^ or Mean (SD)			N (%)^b^ or Mean (SD)		
*Outcome*							
HIV Sexual Risk							
Yes	180 (63.8%)	144 (59.5%)	36 (90.0%)	.0001	152 (60.1%)	28 (96.6%)	<.0001
No	102 (36.2%)	98 (40.5%)	4 (10.0%)		101 (39.9%)	1 (3.4%)	
*Sociodemographic and contextual characteristics*							
Age	44.9 (11.5)	45.2 (11.7)	43.7 (10.6)	0.42	45.2 (11.7)	43.0 (10.1)	0.29
Sex							
Male	182 (64.5%)	159 (65.7%)	23 (57.5%)	0.32	166 (65.6%)	16 (55.2%)	0.27
Female	100 (35.5%)	83 (34.3%)	17 (42.5%)		87 (34.4%)	13 (44.8%)	
Race							
Black	183 (64.9%)	154 (63.6%)	29 (72.5%)	0.55	161 (63.6%)	22 (75.9%)	0.34
White	72 (25.5%)	64 (26.4%)	8 (20.0%)		66 (26.1%)	6 (20.7%)	
Other	27 (9.6%)	24 (9.9%)	3 (7.5%)		26 (10.3%)	1 (3.4%)	
Income in the last month (in dollars)	1070 (5970)	1130 (6430)	670 (1200)	0.31	1090 (6290)	861 (1420)	0.63
Education							
Less than high school	59 (20.9%)	54 (22.3%)	5 (12.5%)	0.32	56 (22.1%)	3 (10.3%)	0.31
High school	136 (48.2%)	116 (47.9%)	20 (50.0%)		121 (47.8%)	15 (51.7%)	
More than high school	87 (30.9%)	72 (29.8%)	15 (37.5%)		76 (30.0%)	11 (37.9%)	
Recent heavy alcohol use (30 days)							
Yes	31 (11.0%)	23 (9.5%)	8 (20.0%)	0.06	23 (9.1%)	8 (27.6%)	0.007
No	251 (89.0%)	219 (90.5%)	32 (80.0%)		230 (90.9%)	21 (72.4%)	
Recent drug use (30 days)							
Yes	74 (26.2%)	58 (24.0%)	16 (40.0%)	0.03	62 (24.5%)	12 (41.4%)	0.05
No	208 (73.8%)	184 (76.0%)	24 (60.0%)		191 (75.5%)	17 (58.6%)	
Recent injection drug use (30 days)							
Yes	6 (2.1%)	4 (1.7%)	2 (5.0%)	0.2	4 (1.6%)	2 (6.9%)	0.12
No	276 (97.9%)	238 (98.3%)	38 (95.0%)		249 (98.4%)	27 (93.1%)	
Recent incarceration (past 2 years)							
Yes	141 (50.0%)	118 (48.8%)	23 (57.5%)	0.39	125 (49.4%)	16 (55.2%)	0.57
No	141 (50.0%)	124 (51.2%)	17 (42.5%)		128 (50.6%)	13 (44.8%)	
Have a mental health diagnosis							
Yes	155 (55.0%)	131 (54.1%)	24 (60.0%)	0.61	139 (54.9%)	16 (55.2%)	0.9
No	127 (45.0%)	111 (45.9%)	16 (40.0%)		114 (45.1%)	13 (44.8%)	
Time lapse between surveys (in weeks)	25.8 (2.3)	25.9 (2.2)	25.7 (2.8)	0.67	25.8 (2.1)	25.9 (3.2)	0.86

a For categorical variables, p-values are from χ^2^ tests. For continuous variables, p-values are from t-tests.

b Percentages present are column percentages.
